# MMP Mediated Degradation of Type IV Collagen Alpha 1 and Alpha 3 Chains Reflects Basement Membrane Remodeling in Experimental and Clinical Fibrosis – Validation of Two Novel Biomarker Assays

**DOI:** 10.1371/journal.pone.0084934

**Published:** 2013-12-23

**Authors:** Jannie Marie Sand, Lise Larsen, Cory Hogaboam, Fernando Martinez, MeiLan Han, Martin Røssel Larsen, Arkadiusz Nawrocki, Qinlong Zheng, Morten Asser Karsdal, Diana Julie Leeming

**Affiliations:** 1 Fibrosis Biology and Biomarkers, Nordic, Bioscience, Herlev, Denmark; 2 Department of Pathology, University of Michigan, Ann Arbor, Michigan, United States of America; 3 Division of Pulmonary and Critical Care Medicine, University of Michigan, Ann Arbor, Michigan, United States of America; 4 Faculty of Health Science, University of Southern Denmark, Odense, Denmark; 5 Nordic Bioscience Beijing, Beijing, China; National Center for Scientific Research Demokritos, Greece

## Abstract

**Objectives:**

Fibrosis is characterized by excessive tissue remodeling resulting from altered expression of various growth factors, cytokines and proteases. We hypothesized that matrix metalloproteinase (MMP) mediated degradation of type IV collagen, a main component of the basement membrane, will release peptide fragments (neo-epitopes) into the circulation. Here we present the development of two competitive enzyme-linked immunosorbent assays (ELISAs) for assessing the levels of specific fragments of type IV collagen α1 (C4M12a1) and α3 (C4M12a3) chains in serum as indicators of fibrosis.

**Methods:**

Fragments of type IV collagen cleaved *in* *vitro* by MMP-12 were identified by mass spectrometry, and two were chosen for ELISA development due to their unique sequences. The assays were evaluated using samples from a carbon tetrachloride (CCl_4_) rat model of liver fibrosis and from patients with idiopathic pulmonary fibrosis (IPF) or chronic obstructive pulmonary disease (COPD).

**Results:**

Two technically robust ELISAs were produced using neo-epitope specific monoclonal antibodies. Mean serum C4M12a1 levels were significantly elevated in CCl_4_-treated rats compared with controls in weeks 12, 16, and 20, with a maximum increase of 102% at week 16 (p < 0.0001). Further, C4M12a1 levels correlated with the total collagen content of the liver in CCl_4_-treated rats (r = 0.43, p = 0.003). Mean serum C4M12a3 levels were significantly elevated in patients with mild, moderate, and severe IPF, and COPD relative to healthy controls, with a maximum increase of 321% in COPD (p < 0.0001).

**Conclusions:**

Two assays measuring C4M12a1 and C4M12a3 enabled quantification of MMP mediated degradation of type IV collagen in serum. C4M12a1 was elevated in a pre-clinical model of liver fibrosis, and C4M12a3 was elevated in IPF and COPD patients. This suggests the use of these assays to investigate pathological remodeling of the basement membrane in different organs. However, validations in larger clinical settings are needed.

## Introduction

Fibrosis is thought to be the result of an abnormal response to persistent or recurrent injury to epithelial cells [1]. It is characterized by fibroblast proliferation and differentiation, and the excessive production of extracellular matrix (ECM) proteins, especially types I and III collagen, that accumulate in the extracellular space [2–4]. In normal tissue the balance between formation and degradation of ECM proteins is strictly controlled to maintain the tissue structure and function. However, in a pathological state such as fibrosis the balance can be disrupted, resulting in excessive accumulation or degradation of proteins. Matrix metalloproteinases (MMPs) have been described as playing an important role in the pathogenesis of fibrosis, both by degrading ECM proteins and activating various signaling molecules [5–8]. A number of conditions lead to such an uncontrolled tissue remodeling, including hepatitis C virus infection and alcoholic liver disease which affect the ECM of the liver, and idiopathic pulmonary fibrosis (IPF) and chronic obstructive pulmonary disease (COPD) which disrupt the ECM of the lung. In each of these conditions, the abnormal ECM remodeling manifests as local fibrosis in the given organ. 

The basement membrane (BM) is a specialized form of ECM that functions as a scaffold for epithelial and endothelial cells, a barrier between tissues, and a substrate for cellular interactions [9,10]. The main components of the BM are type IV collagen and laminin that are found in distinct networks linked together by nidogen and heparin sulfates [11,12]. Type IV collagen is made up of six distinct alpha chains, α1-6(IV), that form the heterotrimers α1α1α2, α3α4α5, and α5α5α6 which are selectively expressed in the mammalian BMs [13,14]. During fetal development, α1α1α2 networks, which are present in all BMs, are partly replaced by other heterotrimers in selected tissues [15,16]. The α3α4α5 network has mainly been identified in lung, kidney, testis, cochlea, and eye, whereas the α5α5α6 network has been located in skin, smooth muscle cells, esophagus, and Bowman’s capsule of the kidney [10,15]. It has been speculated that the replacement of the fetal α1α1α2(IV) network with α3α4α5(IV) in kidney and lungs serves to protect the BM from proteolytic degradation at exposed sites of filtration in the glomeruli and gas exchange in the alveoli [17]. The important structural role of type IV collagen may be illustrated by the clinical manifestations of Alport’s syndrome and Goodpasture’s syndrome. In both disorders, damage to type IV collagen due to mutations or immune attacks lead to kidney and/or lung failure [16]. 

The tissue injuries that eventually lead to fibrosis induce the secretion of various pro-fibrotic and pro-inflammatory mediators including interleukins, tumor necrosis factor (TNF)-α, and transforming growth factor (TGF)-β [18]. Among the effects of this is a local increase in protease secretion, including MMP-2 and MMP-9 [19], and an influx of macrophages to the site of injury, secreting the macrophage metalloelastase MMP-12 [20]. MMP-2, MMP-9, and MMP-12 degrades type IV collagen, thus disrupting the BM and enabling access to the fibrotic tissue for incoming fibroblasts and macrophages [21–24]. Degradation of type IV collagen results in the release of protein fragments, referred to as neo-epitopes or protein fingerprints, into the circulation where they can be quantified as biochemical markers of BM turnover [25]. Disease assessments are more readily made by measuring biochemical marker levels in serum than by invasive and time-consuming techniques such as biopsies and imaging [26]. The assessment of a specific posttranslational modification (PTM) to a protein may provide unique information, giving a more detailed description of events than total protein measurements. For example, type I collagen may be isomerized, cross-linked, and proteolytically degraded, and by assessing these different PTMs, information on such characteristics as bone formation (pro-peptide) [27], bone resorption (isomerized, cross-linked and cathepsin K-degraded) [28] and soft tissue turnover (MMP-degraded) [29] may be obtained. Assessments of total type I collagen content would not offer this sort of information, but merely estimate the current level of total protein. 

Here we present the development and validation of two competitive enzyme-linked immunosorbent assays (ELISAs) that target the α1 and α3 chains of type IV collagen in order to evaluate fibrosis of different organs. The C4M12a1 ELISA targets a neo-epitope positioned in the ubiquitously found α1(IV) chain and the C4M12a3 ELISA is directed at a neo-epitope in the α3(IV) chain that shows restricted tissue distribution. Both neo-epitopes are thought to be released into the circulation by proteolytic degradation by MMPs, and can thus be measured in serum. Due to the chain position of the neo-epitopes, C4M12a1 was evaluated for its ability to describe remodeling of the BM during liver fibrosis, while C4M12a3 was evaluated for its relationship to the lung disorders IPF and COPD. 

## Materials and Methods

### Ethics statement

The mouse work was approved by the Beijing laboratory animal administration office under approval number 200911250. The rat study was approved by the Animal Experimentation Ethics Committee of the University of Barcelona (approval #B-NNP-233/09) and was performed according to the criteria of the Investigation and Ethics Committee of the Hospital Clinic Universitari (Barcelona, Spain). Human serum samples were obtained as a part of the “Lung Tissue Research Consortium” (LTRC, www.ltrcpublic.com) from patients diagnosed with IPF or COPD who had provided their written informed consent. The institutional review board (IRB) of the University of Michigan Medical School evaluated the study and concluded that due to the proper de-identification of samples and patients by the LTRC, an approval from the IRB was not required for this work. 

### Reagents

All reagents used for the experiments were standard high-quality chemicals from companies such as Merck (Whitehouse Station, NJ, USA) and Sigma Aldrich (St. Louis, MO, USA). The synthetic peptides used for monoclonal antibody production were purchased from the Chinese Peptide Company, Beijing, China.

### *In vitro* cleavage

#### Peptide identification

Purified type IV collagen (cat. no. ab7536, Abcam, Cambridge, UK) was cleaved with active MMP-12 (cat. no. ab54058, Abcam) by mixing 100 µg type IV collagen and 1 µg MMP-12 in MMP buffer (50 mM TRIS, 10 mM CaCl_2_, 200 mM NaCl, 100 µM ZnSO_4_, 0.05% Brij-35, pH 7.5). As control, 100 µg of type IV collagen was mixed with MMP buffer alone. The solutions were incubated for seven days at 37°C. The cleavage reaction was stopped using ethylenediaminetetraacetic acid (EDTA) to a final concentration of 1 µM. 

#### ELISA characterization

Purified type IV collagen (cat. no. ab7536, Abcam) was cleaved with recombinant human MMP-12 (cat. no. 917-MP, R&D systems, Abingdon, Oxfordshire, UK), MMP-2 (cat. no. 444213, Calbiochem, Billerica, MA, USA), MMP-9 (cat. no. 444231, Calbiochem) and pepsin (cat. no. P7012, Sigma Aldrich) as follows: 100 µg type IV collagen was mixed with 200 µg pepsin in 10 mM HCl and incubated at 37°C for 30 minutes. Pepsin digestion was stopped by adjusting the pH to neutral by adding 100 mM NaOH. MMP-12 was activated by incubation in MMP buffer at 37°C for 24 hours. Subsequently, 2 µg MMP-12 was incubated with 50 µg type IV collagen or pepsin-treated type IV collagen in MMP buffer for 48 hours at 37°C. MMP-2 and MMP-9 were activated in 3 mM APMA by incubation at 37°C for 3 hours. Subsequently, 2 µg MMP-2 or MMP-9 was incubated with 50 µg type IV collagen in MMP buffer for 24 hours at 37°C. MMP cleavage was stopped by adding EDTA to a final concentration of 1 µM. Type IV collagen incubated in MMP buffer without added enzymes was used as control. All cleavages were verified by visualization using the SilverXpress® Silver Staining Kit (cat. no. LC6100, Life Technologies, Nærum, Denmark) according to the manufacturer’s instructions.

### Peptide identification

The peptides produced by the MMP-12 cleavage of type IV collagen were purified and desalted using reversed-phase (RP) micro-columns (cat. no. 1-1332-26, Life Technologies) prior to nanoLC-MS-MS analysis as described in the literature [30]. The purified peptides were re-suspended in 100% formic acid, diluted with H_2_O and loaded directly onto an 18 cm RP capillary column using a nano-Easy-LC system (cat. no. LC120, Thermo Fisher Scientific, Hvidovre, Denmark). The peptides were eluted using a gradient from 100% phase A (0.1% formic acid) to 35% phase B (0.1% formic acid, 95% acetonitrile) over 43 minutes directly into a LTQ-Orbitrap XL mass spectrometer (Thermo Fisher Scientific). From each MS scan (Orbitrap) taken at a resolution of 60,000 to detect molecules in the range of 300-1800 Da, the five most abundant precursor ions were selected for fragmentation (collision-induced dissociation). The raw data files were converted to mgf files and searched with Mascot 2.2 using the Proteome Discoverer software (Thermo Fisher Scientific). Peptides with a mascot probability score of p < 0.05 were analyzed further. The six amino acids in the N- or C-terminal of the peptides identified by MS in cleavage samples but not in control samples were each regarded as a neo-epitope generated by the protease. All protease-generated sequences were analyzed for sequence uniqueness and species homology by protein blasting [31]. 

### Peptide conjugation

The peptide conjugation was performed using a Maleimide Activated Immunogen Conjugation Kit (cat. no. K0383, Sigma-Aldrich). Briefly, 2 mg cysteine-containing immunogenic neo-epitope peptides (ILGHVPGMLL-GGC (C4M12a1) or PGDIVFRKGP-GGC (C4M12a3)) containing one free sulfhydryl (-SH) group was mixed in conjugation buffer with 1.8 mg maleimide-activated carrier protein keyhole limpet hemocyanin (KLH), which had an available maleimide group that could react with the -SH-containing peptides . The mix was incubated for 2 hours at room temperature and conjugated products were cleared of EDTA and sodium azide by desalting or dialysis for two days. For the biotin-conjugated peptides, the biotin-conjugated lysine was added in the solid-phase peptide synthesis procedure.

### Monoclonal antibody development

Four to six-week-old Balb/C mice were immunized subcutaneously with 200 µl emulsified antigen and 50 µg of the C4M12a1 (ILGHVPGMLL-GGC-KLH) or C4M12a3 (PGDIVFRKGP-GGC-KLH) peptide using Freund’s incomplete adjuvant. Immunizations were performed every 2^nd^ week until stable sera titer levels were reached. The mouse with highest serum titer was selected for fusion. The mouse was rested for one month and then boosted intravenously with 50 µg C4M12a1 or C4M12a3 peptide in 100 µl 0.9% sodium chloride solution three days before isolation of the spleen for cell fusion. The fusion procedure was performed as previously described [32]. Briefly, mouse spleen cells were fused with SP2/0 myeloma fusion partner cells. The resulting hybridoma cells were cloned using a semi-solid medium method, transferred into 96-well microtiter plates for further growth and incubated in a CO_2_ incubator. Standard limited dilution was used to promote monoclonal growth. 

### Clone characterization

Native reactivity and peptide affinity of the monoclonal antibodies were evaluated by displacement of native samples (human, rat, and mouse serum, plasma, and urine) in a preliminary indirect ELISA using biotinylated peptides (ILGHVPGMLL-K-biotin (C4M12a1) or PGDIVFRKGP-K-biotin (C4M12a3)) on streptavidin-coated microtiter plates and the supernatant from the growing monoclonal hybridoma. Specificities of the clones to the free peptide (ILGHVPGMLL or PGDIVFRKGP), a nonsense peptide, and an elongated peptide (EILGHVPGMLL or PPGDIVFRKGP) were tested. Isotyping of the monoclonal antibodies was performed using a SBA Clonotyping System-HRP kit (cat. no. 5300-05, Southern Biotech, Birmingham, AL, USA). The monoclonal antibody was purified from collected supernatant of the selected clones using HiTrap protein G columns (cat. no. 17-0404, GE Healthcare Life Science, Little Chalfont, Buckinghamshire, UK) and subsequently labeled with horseradish peroxidase (HRP) using the Lightning link HRP labeling kit (cat. no. 701, Innova Bioscience, Babraham, Cambridge, UK), according to the manufacturer’s instructions. 

### ELISA protocol

The final C4M12a1 and C4M12a3 competitive ELISA procedures were as follows: A 96-well streptavidin-coated microtiter plate (cat. no. 11940279, Roche Diagnostics, Hvidovre, Denmark) was coated with 100 µl biotinylated peptide (ILGHVPGMLL-K-biotin for C4M12a1 and PGDIVFRKGP-K-biotin for C4M12a3) dissolved in coating buffer (50 mM Tris, containing 1% bovine serum albumin, 0.1% Tween-20, and 0.4% bronidox (BTB), pH 8.0 for C4M12a1 and 25 mM PBS-BTB, pH 7.4 for C4M12a3) and incubated for 30 minutes at 20°C. 20 µl standard peptide or sample dissolved in assay buffer (50 mM Tris-BTB, pH 8.0 for C4M12a1 and 25 mM PBS-BTB containing 5% Liquid II, pH 7.4 for C4M12a3) was added to appropriate wells, followed by 100 µl of conjugated monoclonal antibody diluted in assay buffer and incubated 1 hour at 20°C (C4M12a1) or 4°C (C4M12a3). Finally, 100 µl tetramethylbenzinidine (TMB) (cat. no. 4380H, Kem-En-Tec, Taastrup, Denmark) was added and the plate was incubated 15 minutes at 20°C. The TMB reaction was stopped by adding 100 µl stopping solution (1% H_2_SO_4_). All incubation steps were performed in the dark with shaking at 300 rpm and followed by five washes in washing buffer (20 mM Tris, 50 mM NaCl, pH 7.2). The results were analyzed spectrophotometrically at 450 nm with 650 nm as the reference using an ELISA microplate reader (VersaMax, Molecular Devices, Sunnyvale, CA, USA). A standard curve was performed by serial dilution of the standard peptide and plotted using a 4-parametric mathematical fit model. 

### Technical evaluation

Two-fold dilutions of human, rat, and mouse serum and plasma samples were used to determine linearity. Recovery percentages were calculated with the undiluted sample as a reference value. The lower limit of detection (LLOD) was calculated as mean + 3x standard deviation (SD) determined from 21 zero samples (i.e. the assay buffer). The inter- and intra-assay variation was determined by 10 independent runs of eight quality control (QC) human serum samples, with each run consisting of two replicas of double determinations of the samples. Intra-assay variation was calculated as the mean coefficient of variance (CV %) within plates, and the inter-assay variation was calculated as the mean CV % between the 10 individual runs. Spiking recovery was determined by spiking two human serum samples diluted 1:2 with two-fold dilutions of human serum or standard peptide, and calculated as percentage recovery by using the expected concentration as a reference value. Interference by hemoglobin, lipemia, biotin, and human antibodies against mouse antigens by human anti-mouse antibody (HAMA) was determined by adding two-fold dilutions to a serum sample of known concentration. Concentrations started at 0.500 mmol/l hemoglobin, 0.56 mmol/l lipemia, and 160 µg/l biotin. A human serum sample with high HAMA content was added to a normal human serum sample in two-fold dilutions. Recovery percentage was calculated with the normal serum sample as a reference value. The analyte stability was determined for three human and three rat serum samples in four freeze/thaw cycles. Recovery was calculated with the first cycle as a reference value.

### ELISA characterization

The ELISAs were evaluated using the following samples: cleaved type IV collagen, a synthetic peptide with an elongated C4M12a1 or C4M12a3 amino acid sequence (EILGHVPGMLL or PPGDIVFRKGP, respectively), and a synthetic peptide with a nonsense sequence (PGPPGIVIGT). To determine the background in the system, the assays were tested using a nonsense biotinylated peptide (biotin-K-PGPPGIVIGT). Reactivity to synthetic peptides of both the human (PGDIVFRKGP) and rat (PGDTVFQPGP) sequence was compared in the C4M12a3 ELISA to ensure that the assay could be used for measurements in both human and rat matrices.

### Carbon tetrachloride-induced liver fibrosis in rats

The study included three-month-old male Wistar rats (Charles-River, Saint-Aubin les Elseuf, France), of which 52 were treated with carbon tetrachloride (CCl_4_) to induce liver fibrosis and 28 were used as controls. Complete details of the study are described elsewhere [33], and the induction of liver fibrosis was performed as previously described [34]. Briefly, CCl_4_ was administered by inhalation twice weekly, and phenobarbital (0.3 g/l) was added to the drinking water. Control rats received phenobarbital only. Animals were stratified into groups receiving 8, 12, 16, or 20 weeks of treatment (n = 13 for CCl_4_ and n = 7 for control for each time point). Prior to termination with isofluorane, blood was collected from fasted rats and allowed to clot at room temperature before centrifugation at 2500 rpm for 10 min. Serum was collected and stored at -80°C for later biomarker assessments. Samples were diluted 1:4 for measurements in the C4M12a1 ELISA. Liver samples were obtained from the middle liver lobe and fixed in 10% buffered formalin for histology analysis. 4 µm thick rat liver sections were stained in 0.1% Sirius red F3B in saturated picric acid (Sigma-Aldrich). The amount of fibrosis was expressed as a percentage of the total liver area by analyzing 36 liver sections per animal and subtracting the vascular luminal area [35]. Results were presented as the mean value of each group. 

### IPF and COPD patient cohort

Serum samples from patients diagnosed with IPF (n = 30) or COPD (n = 10), were obtained as a part of the LTRC. IPF patients were stratified according to forced vital capacity (FVC): mild IPF (FVC > 80%, n = 10), moderate IPF (FVC = 50-80%, n = 10), and severe IPF (FVC < 50%, n = 10). All COPD patients had forced expiratory volume in one second (FEV_1_) > 80% of expected, defined as mild COPD. Limited data were available from this cohort, only including FVC or FEV_1_. Control serum samples (n = 23) were collected from fasting healthy individuals. Samples were diluted 1:2 for measurements in the C4M12a3 ELISA.

### Statistical analyses

The serum levels of C4M12a1 were assessed in control and CCl_4_-treated rats and analyzed using unpaired samples t-test with Welch’s correction. Data were shown as mean ± standard error of mean (SEM). Correlations between serum C4M12a1 levels and amount of liver fibrosis were analyzed using Spearman rank correlation coefficient. Serum C4M12a3 levels in COPD patients, IPF patients and healthy controls were logarithmically transformed to obtain normality and symmetry of variance. Comparisons of levels were analyzed using ANOVA with Dunn’s multiple comparisons test and data were shown as geometric mean ± SEM on the back-transformed data P values less than 0.05 were considered statistically significant. Statistical analyses were performed using GraphPad Prism v6 (GraphPad Software, La Jolla, CA, USA).

## Results

### Selection of peptides for assay development

**Figure 1:**
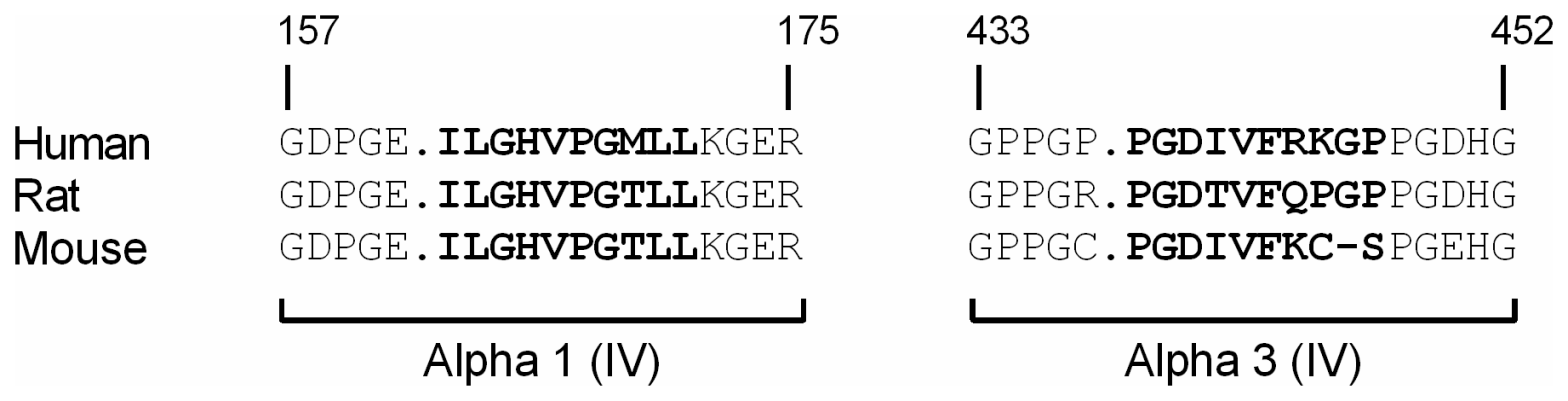
Alignment of type IV collagen α1 and α3 chains. The sequence of the type IV collagen α1 chain (left) and α3 chain (right) were aligned for the species human, rat, and mouse. Bold types indicate the target sequence, while dots indicate the MMP-12 cleavage site recognized by the monoclonal antibody employed in the respective assay (left: C4M12a1, right: C4M12a3).

Fragments generated *in vitro* by cleavage of type IV collagen with MMP-12 were identified with a statistically significant MASCOT score (p < 0.05). All MMP-12 generated peptides were tested for homology to other proteins and species, and two sequences were selected for immunizations since blasting showed that these sequences were unique to type IV collagen. The sequence 162'.ILGHVPGMLL'171 (C4M12a1) in the human α1(IV) chain was 90% homologous to rat and mouse species (162'.ILGHVPGTLL'171), and the six amino acid target sequence showed 100% homology (Figure 1, left). The sequence 438'.PGDIVFRKGP'447 (C4M12a3) was found in the human α3(IV) chain and was 70% homologous to mouse (438'.PGDIVFKCSP'447) and rat (438'.PGDTVFQPGP'447) (Figure 1, right). However, the six amino acid target sequence PGDIVF was 100% homologous to the corresponding mouse sequence and 83% to the corresponding rat sequence.

### Characterization of antibody clones

**Figure 2:**
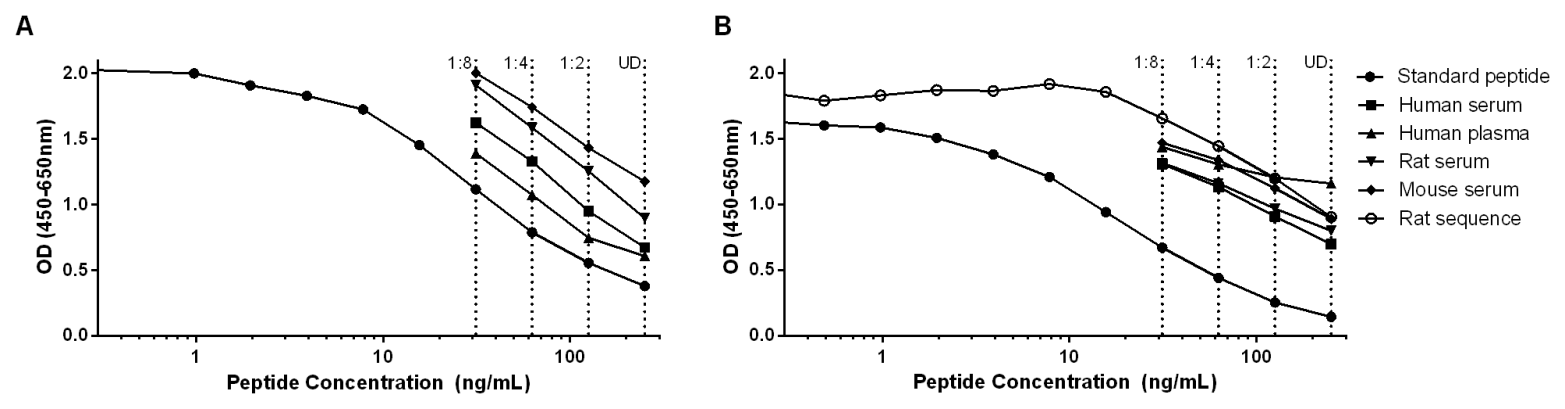
Standard curves and native reactivity. Typical standard curves and native reactivity against human, rat, and mouse material diluted to 1:8 as indicated for the assays C4M12a1 (A) and C4M12a3 (B). The reactivity of the C4M12a3 ELISA to the rat sequence (PGDTVFQPGP) was also tested (B). Signals are shown as optical density (OD) at 450 nm, subtracting the background at 650 nm, as a function of standard peptide concentration. UD: undiluted sample.

The monoclonal antibody with the best native reactivity, peptide affinity, and stability in each assay was chosen from the antibody-producing clones generated after fusion between mouse spleen cells and myeloma cells. The clones selected were of the IgG1 subtype and the antibodies showed reactivity to healthy human, rat, and mouse serum, as well as human plasma EDTA (Figure 2). The assay measuring C4M12a3 showed reactivity to a synthetic peptide of the rat sequence, but had a higher affinity for the standard peptide with the human sequence (Figure 2B), indicating the preferred use of this assay with human samples.

### Technical evaluation

A typical standard curve for each assay, showing a 4-parametric fit, is presented in Figure 2. The LLOD was 0.34 ng/mL for the C4M12a1 ELISA and 0.92 ng/mL for the C4M12a3 ELISA. Both assays were sensitive up to marker concentrations of 200 ng/mL. The C4M12a3 assay showed dilution recoveries close to 100% from undiluted to 1:8 for human, rat, and mouse serum (Table 1). For the C4M12a1 ELISA, dilution recoveries of human serum were close to 100% for undiluted to 1:8, while rat serum could only be diluted to 1:4 since further dilutions gave marker levels below LLOD. C4M12a1 levels in mouse serum were low and the marker was only detectable in undiluted matrix and at a dilution of 1:2. Human plasma EDTA needed to be diluted at least 1:2 for both assays to obtain linearity. At the stated dilutions, all recoveries were within the acceptable range of 100±20% (Table 1). Intra- and inter-assay variations were 2.8% and 9.8%, respectively for C4M12a1 and 9.6% and 11.5%, respectively for C4M12a3 (Table 2). The analyte stability was acceptable for four freeze/thaw cycles of human and rat serum, being within 100±20% for both assays (Table 2). Spiking recovery for standard peptide in serum and serum in serum were acceptable, being within 100±20% for both assays (Table 2). Interference tests using hemoglobin, lipemia, biotin, and HAMA showed that these well-known interference substances did not generate interference when added to the assays at high concentrations (Table 2). 

**Table 1:** Linearity of the C4M12a1 and C4M12a3 assays.

**Assay**	**C4M12a1**				**C4M12a3**			
**Sample**	**HS**	**HP**	**RS**	**MS**	**HS**	**HP**	**RS**	**MS**
**Undiluted**	100	-	100	100	100	-	100	100
**1:2**	106	100	95	101	100	100	99	104
**1:4**	96	98	86	-	100	110	103	96
**1:8**	90	99	-	-	106	119	101	92
**Mean**	**98**	**99**	**93**	**101**	**102**	**110**	**101**	**98**

Percentage dilution recovery for representative serum and plasma samples, calculated relative to the undiluted sample (or 1:2 for human plasma). HS: human serum. HP: human plasma EDTA. RS: rat serum. MS: mouse serum.

**Table 2:** Technical characterization of the C4M12a1 and C4M12a3 assays.

**Technical Characteristics**	**C4M12a1 (percent)**	**C4M12a3 (percent)**
Intra-assay Variation**^*a*^**	2.8	9.6
Inter-assay Variation**^*a*^**	9.8	11.5
Analyte Stability**^*b*^**	96	85
Spiking Recovery Peptide in Serum**^*c*^**	86	118
Spiking Recovery Serum in Serum**^*c*^**	104	110
Hemoglobin Interference**^*d*^**	97	97
Lipemia Interference**^*d*^**	91	95
Biotin Interference**^*d*^**	91	120
HAMA Interference**^*d*^**	120	101

^a^ The intra- and inter-assay variation was determined by repeated measurements of eight quality control human serum samples. Variation was calculated as the mean variation between 10 individual determinations of each sample.

^b^ The recovery percentage of four freeze/thaw cycles was calculated with cycle one as a reference value for three human and three rat serum samples.

^c^ Two-fold dilutions of standard peptide or human serum were spiked into a human serum sample diluted 1:2. The recovery percentage was calculated as the measured concentration relative to expected concentrations of peptide/serum in serum.

^d^ Hemoglobin (0.5mmol/l), lipemia (0.56mmol/l), biotin (160µg/l), or a human serum sample containing high concentrations of human anti-mouse antibodies (HAMA) was diluted two-fold and added to a human serum sample of known concentration. Mean recovery percentage was calculated with the normal serum sample as a reference value.

### Assay characterization

**Figure 3:**
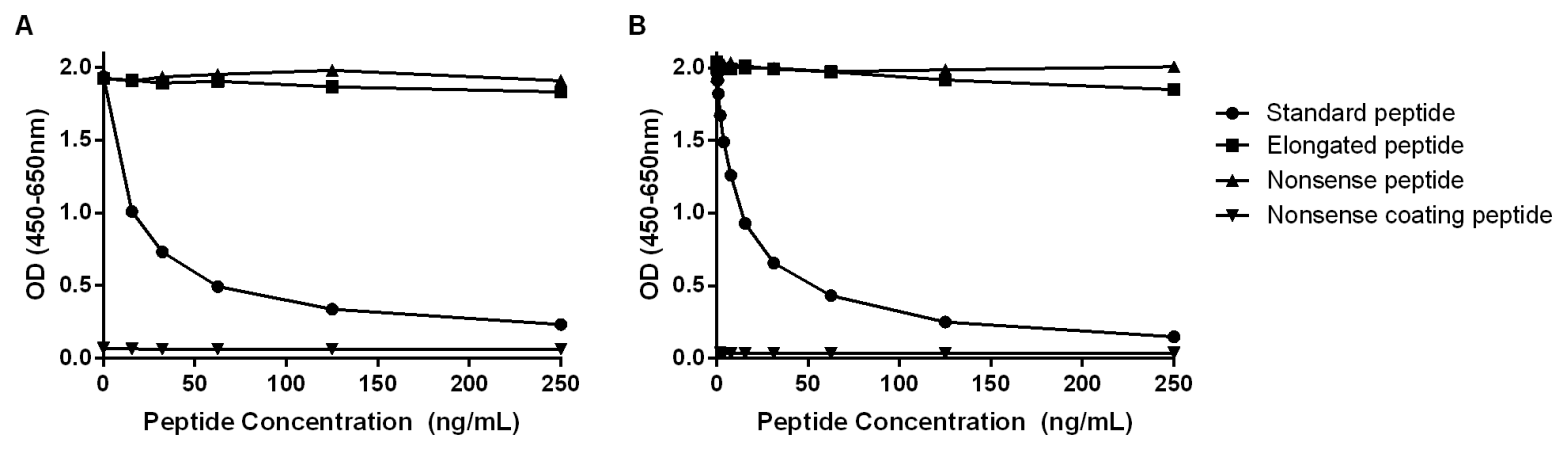
Assay specificity. Reactivity to the standard peptide (A: ILGHVPGMLL; B: PGDIVFRKGP), the elongated peptide (A: EILGHVPGMLL; B: PPGDIVFRKGP) and a nonsense peptide (A + B: PGPPGIVIGT) was tested for the assays C4M12a1 (A) and C4M12a3 (B). The background signal from the system was tested using a nonsense coating peptide (A+B: biotin-K-PGPPGIVIGT). Signals are shown as optical density (OD) at 450 nm, subtracting the background at 650 nm, as a function of standard peptide concentration.

**Figure 4:**
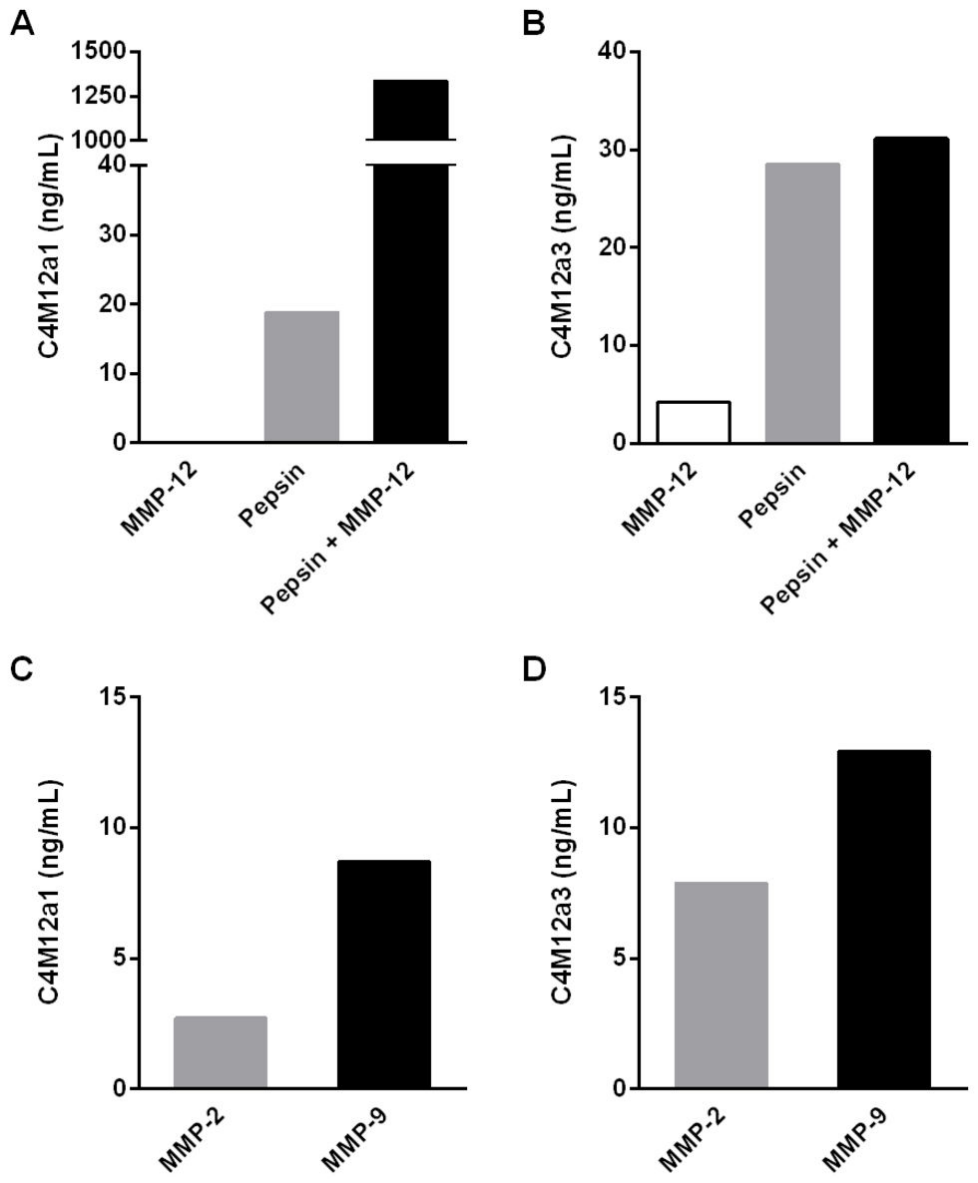
Reactivity to cleaved type IV collagen. C4M12a1 (A and C) and C4M12a3 (B and D) marker levels were assessed in samples of purified type IV collagen cleaved *in* *vitro* by MMP-12, pepsin, and MMP-12 in combination with pepsin (A and B), as well as MMP-2 and MMP-9 (C and D). Data were normalized by subtraction of intact type IV collagen background.

The analytes detected by the C4M12a1 and C4M12a3 ELISAs were characterized by testing reactivity towards synthetic peptides, as well as cleaved type IV collagen. The assays did not show reactivity towards an elongated synthetic peptide or a nonsense synthetic peptide (Figure 3), indicating neo-epitope specificity for both antibodies. Furthermore, no background signal was detected in each assay tested against a nonsense coating peptide (Figure 3). The neo-epitope specificity of the antibody employed within the assays was further characterized by measurements of analytes in samples of type IV collagen cleaved by MMP-2, MMP-9, and MMP-12. Cleavages by pepsin alone and in combination with MMP-12 were also tested to mimic a possible pre-digestion performed by other proteases in tissue (Figure 4). The level of C4M12a1 was highly increased to 1335 ng/mL above intact type IV collagen when cleaved by pepsin and MMP-12 in combination. Pepsin cleavage promoted an increase of 19 ng/mL in the C4M12a1 level, but MMP-12 cleavage did not show levels above the intact protein (Figure 4A). Levels of C4M12a3 were increased to 4 ng/mL above the level of intact type IV collagen when cleaved by MMP-12, while pepsin alone and in combination with MMP-12 produced similar increases of 29 and 31 ng/mL, respectively (Figure 4B). C4M12a1 levels of 3 ng/mL and 9 ng/mL above the intact protein was produced by MMP-2 and MMP-9 cleavage, respectively (Figure 4C), and C4M12a3 levels were elevated with 8 ng/mL and 13 ng/mL, respectively (Figure 4D). All measurements of intact type IV collagen were low and at a level with the background measured in buffer alone.

### C4M12a1 as a marker of liver fibrosis in rats

**Figure 5:**
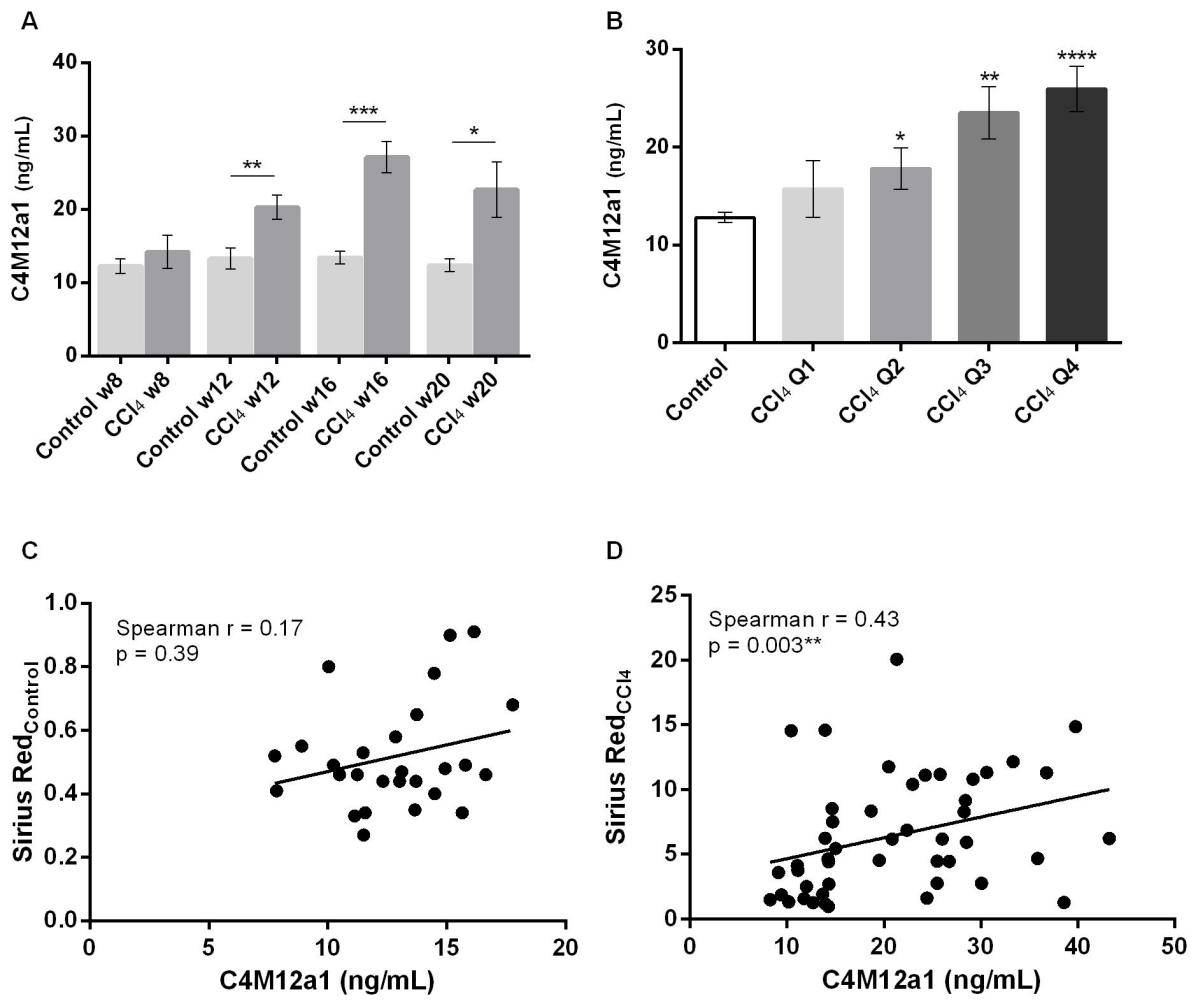
Serum C4M12a1 levels in carbon tetrachloride-treated rats. *A*: Serum C4M12a1 was assessed in control rats and carbon tetrachloride (CCl_4_)-treated rats at termination at week 8, 12, 16, and 20. Results are shown as mean ± SEM. *B*: Serum C4M12a1 levels of CCl_4_-treated rats were stratified into quartiles according to total collagen content of the liver as determined by Sirius red staining. Results are shown as mean ± SEM. *C+D*: Correlation of total collagen content of the liver (Sirius red) with serum C4M12a1 levels for controls (C) and CCl_4_-treated rats (D). Spearman r: Spearman correlation coefficient. Significance levels: *: p < 0.05, **: p < 0.01, ***: p < 0.001, ****: p < 0.0001.

Mean levels of serum C4M12a1 were significantly elevated at weeks 12, 16 and 20 in CCl_4_-treated rats compared with vehicle-treated controls (week 12: 53% increased, p = 0.006; week 16: 102% increased, p < 0.0001, week 20: 83% increased, p = 0.03) (Figure 5A). CCl_4_-treated animals were stratified into quartiles according to the total amount of collagen in the liver as determined by Sirius red staining. Mean serum C4M12a1 levels were significantly elevated in quartiles 2, 3, and 4 (Q2: 39% increased, p = 0.04; Q3: 84% increased, p = 0.002; Q4: 103% increased, p < 0.0001) compared with control animals (Figure 5B). A correlation between serum C4M12a1 levels and total collagen content of the liver was seen in CCl_4_-treated rats (r = 0.43, p = 0.003) (Figure 5D), but not in control rats (r = 0.17, p = 0.39) (Figure 5C). 

### Evaluation of C4M12a3 in IPF and COPD patients

**Figure 6:**
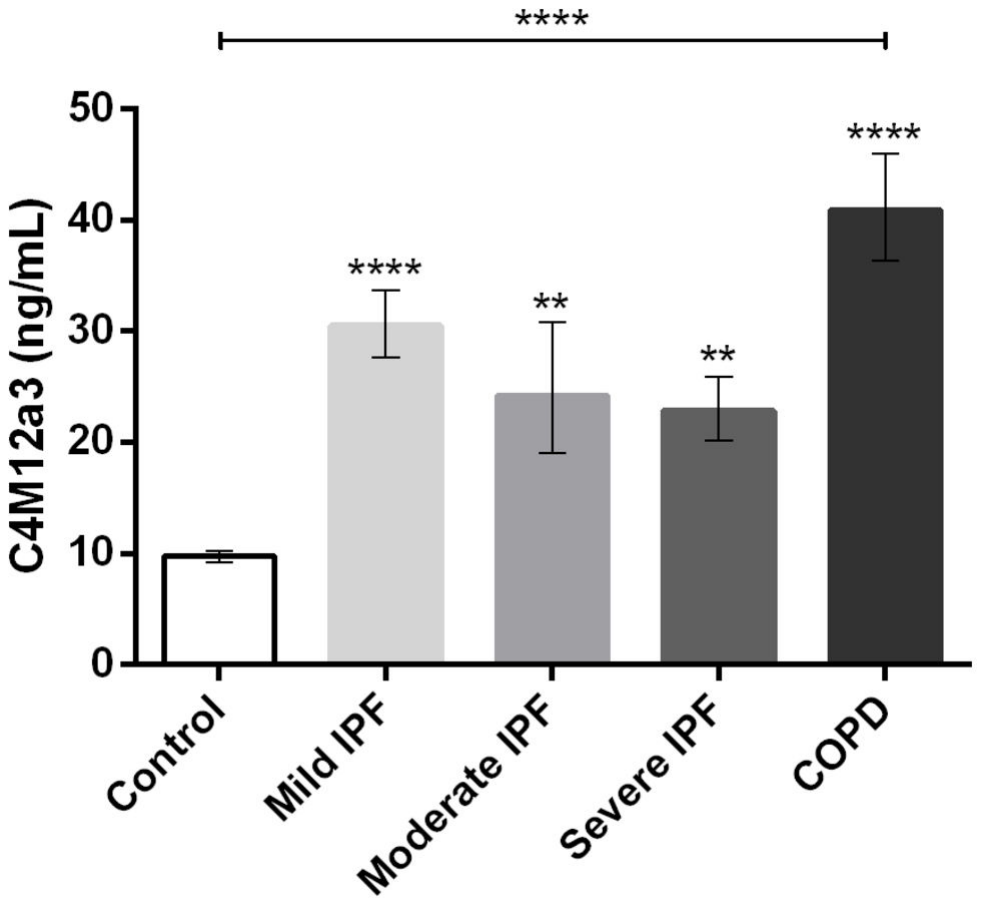
Serum C4M12a3 levels in IPF and COPD patients. Serum C4M12a3 was assessed in healthy controls (n = 23) and patients with mild IPF (n = 10), moderate IPF (n = 10), severe IPF (n = 10), and mild COPD (n = 10). Results are presented as geometric mean ± SEM. Significance levels: *: p< 0.05, **: p < 0.01, ***: p < 0.001, ****: p < 0.0001.

Mean serum C4M12a3 levels were compared in patients with mild IPF, moderate IPF, severe IPF, or mild COPD and in healthy controls, and showed an overall statistical significant difference between groups with p < 0.0001 (Figure 6). All patient groups had significantly elevated serum C4M12a3 levels as compared with healthy controls (mild IPF: 214% increased, p < 0.0001; moderate IPF: 149% increased, p < 0.01; severe IPF: 135% increased, p < 0.01; COPD: 321% increased, p < 0.0001). Furthermore, a 34% increase in serum C4M12a3 levels in patients with mild IPF as compared with severe IPF was seen, but this difference was not statistically significant.

## Discussion

We have described the development of two novel competitive ELISAs for the assessment of MMP mediated degradation of type IV collagen in serum. We found that a general BM remodeling marker, C4M12a1, was correlated to the amount of liver fibrosis in a gold standard animal model of liver fibrosis. A more lung-specific marker of BM remodeling, C4M12a3, was analyzed in a cohort of IPF and COPD patients, showing that this marker was elevated in serum of both IPF and COPD patients compared with healthy controls.

The newly developed C4M12a1 and C4M12a3 assays showed a high reactivity towards their respective synthetic peptide sequences and high native reactivity to human, rat, and mouse serum as well as human plasma. The assays were technically robust, showing low values of LLOD, intra- and inter-assay variation, and interference, and acceptable dilution recovery, spiking recovery, and analyte stability. The finding that neither assay detected the elongated peptide nor a nonsense peptide indicated that the monoclonal antibodies employed in these assays were specific towards the cleavage site at amino acid 162 located in the 7S domain of α1(IV) (C4M12a1) and amino acid 438 located in the triple helical domain of α3(IV) (C4M12a3). This was supported by the detection of increased marker levels after *in vitro* cleavage of type IV collagen with MMP-12 and/or pepsin, as well as MMP-2 and MMP-9. These are important characteristics of the assays, indicating that these monoclonal antibodies are specific for degraded type IV collagen and do not react with the intact protein. The C4M12a1 and C4M12a3 neo-epitopes were both released by MMP-2 and MMP-9, gelatinases with a high specificity for type IV collagen and known to be up-regulated in fibrosis [6,8]. C4M12a1 was not released by MMP-12 cleavage alone, but only in combination with pepsin. This may be explained by the complex nature of the BM [10], rendering a single protease unable to degrade its components. C4M12a3 however, was generated by MMP-12 alone, and the addition of pepsin further increased the release of the C4M12a3 neo-epitope. Type IV collagen is located in a complex network closely connected to other BM proteins, and shows numerous interactions with both cells and proteins [9]. Indeed, pepsin pre-treatment is commonly used to solubilize type IV collagen for extractions and prior to cleavage procedures [24,36,37]. Thus, cleavage by another protease may be needed for MMP-12 to release neo-epitopes from the BM. MMP-2 has been shown to alter the type IV collagen cleavage pattern of MMP-9 [23], making it plausible that another protease may work in concert with MMP-12. The degradation of many multivalent proteins such as collagens depends on enzymes to generate the first cut in the protein. Such a first cut results in uncoiling of the triple helical structure which allows for subsequent cleavage by additional enzymes. This phenomenon is well documented in type I collagen, where interstitial collagenases (MMP-1 or -13) or cathepsin K are essential for its degradation by producing a first cut in the helical region of the protein [38–40]. Subsequently, type I collagen is accessible for other enzymes such as MMP-2 and -9 which may degrade it further [41,42]. In a similar fashion, type IV collagen may be cleaved, albeit without release of the neo-epitope as this is further bound to the protein complex. 

Other assays have previously been developed for the assessment of type IV collagen in serum. Both radioimmunoassays and enzyme immunoassays using polyclonal or monoclonal antibodies have been described [43–51]. The novel competitive ELISAs presented here are unique as they employ a monoclonal antibody to assess a neo-epitope that can only be recognized following cleavage at specific locations in the α1(IV) and α3(IV) chains. Further, since the C4M12a1 and C4M12a3 peptides are generated by MMPs which are up-regulated in fibrosis, the serum levels reflect pathological degradation of type IV collagen and thus BM remodeling. To our knowledge, only one other assay has been described that uses the neo-epitope approach to detect degraded type IV collagen [48]. This assay recognizes a fragment of α1(IV) located between the NC1 and triple helical domains generated by MMP-9. Although similar to the C4M12a1 neo-epitope described here, it is not generated by MMP-2 or MMP-12, and the location in the α1 chain is different. A recent review describes the importance of knowing exactly what is recognized in an assay and highlights the diverse information that can be achieved by assessing different epitopes of the same protein [52]. Thus, these two assays may provide different information on the type IV collagen remodeling. For example, the cleavage sites may be exposed by different routes due to the position in the 7S domain versus the NC1/triple helical domain, thus reflecting separate disease mechanisms. 

The α1(IV) chain is ubiquitously present in the mammalian body, and thus the C4M12a1 neo-epitope measured systemically may originate from a number of organs. Serum C4M12a3 may also originate from several locations, but the distribution of the α3(IV) chain is more restricted and this assay may enable more tissue specific assessments. However, we believe that the C4M12a1 and C4M12a3 serum levels are remarkably higher during pathological remodeling than in healthy individuals, allowing us to overlook the background signal produced by normal tissue remodeling in other organs. In accordance with this, we have in the present study shown that the serum levels of these markers were significantly lower in healthy individuals than during the pathological state of liver and lung fibrosis. 

The value of type IV collagen markers in the assessment of liver and lung fibrosis have previously been described [46–48,53–55]. We found that mean serum C4M12a1 levels were significantly elevated in rats treated with CCl_4_ as compared with controls at 12, 16, and 20 weeks of treatment. Interestingly, serum C4M12a1 levels correlated with the amount of liver fibrosis in the CCl_4_-treated rats, indicating that marker levels were related to the severity of liver fibrosis. This correlation has previously been shown for type IV collagen-derived peptides by Matsumoto et al. [56] and Veidal et al. [48]. The healthy liver contains very limited amounts of BM, but during fibrogenesis a BM-like structure is formed in the space of Disse [57]. When this structure is degraded as part of the fibrotic response, the release of BM fragments from the liver is greatly enhanced, making C4M12a1 a relevant marker of the severity of liver fibrosis. The C4M12a3 marker was not measured in the CCl_4_ rat study since i) the antibody affinity was lower for the C4M12a3 sequence found in rats than for that in humans, and ii) the α3(IV) chain has restricted distribution and is generally found in lung and kidney, but not in the liver [15,58]. Serum C4M12a3 was assessed in a small cohort of patients with IPF and mild COPD, and levels were found to be significantly elevated in all patient groups when compared with healthy controls. COPD patients in general have a high level of protein degradation in the lungs [59,60], as was reflected by their high serum C4M12a3 levels in this study. IPF patients have a particular high degree of lung fibrosis [61], and our results indicate that serum C4M12a3 was related to lung fibrosis. A tendency towards higher serum C4M12a3 levels in patients with mild IPF relative to severe IPF was noted, indicating that serum C4M12a3 is not related to disease severity. In the most active stages of IPF, for example during disease initiation and exacerbations, a more pronounced inflammatory response is seen than in stages of established fibrosis [62]. Thus, this novel marker of type IV collagen turnover, C4M12a3, may reflect disease activity rather than severity. The balance between protein formation and degradation is crucial for upholding the structure and function of the ECM. The ECM protein turnover rate is elevated during fibrosis, showing both increased formation and degradation, which results in a net accumulation of proteins in the extracellular space. Thus, a marker of protein degradation may be better suited for describing fibrosis activity than severity. C4M12a3 may prove interesting in the characterization of lung diseases to provide insight into the BM turnover rate at various disease stages, and it might be useful to identify subgroups of patients showing an increased inflammatory response. The finding that serum C4M12a3 is related to lung diseases is in line with the results of other groups, showing that serum levels of the 7S domain were elevated in IPF patients relative to healthy controls [54,55]. 

The C4M12a1 and C4M12a3 assays may be more useful to characterize fibrosis activity than measures of total type IV collagen due to the dependency on protease activity incorporated into these markers. Assays such as these, that quantify PTMs representing key events in pathology, may have the potential to separate patients according to burden of disease, disease subtype, treatment possibilities, or prognosis, thus enabling personalized health care by using a simple blood sample [25,52]. The C4M12a1 and C4M12a3 ELISAs may prove interesting as tools to characterize patient subgroups according to their MMP mediated BM remodeling. Patient stratification into groups of responders and non-responders of a given intervention based on marker levels may optimize personal health care. Furthermore, a fast responding serum marker reflecting changes in the ECM remodeling will make the evaluation of novel drug candidates more efficient and clinical trials more cost-effective. However, the present results on C4M12a1 remain pre-clinical and thus needs to be verified in human subjects. Further, the use of C4M12a3 has been evaluated in de-identified samples from a small cohort of IPF and COPD patients, limiting the analyses possible for this study. Many markers of lung disorders are influenced by smoking status, and further analyses in well-characterized studies are needed to identify any effects of confounding factors in this assay. Although the results presented here are preliminary, these assays have shown technical stability and promising value in pre-clinical and clinical settings, encouraging their future use in assessing fibrosis of different organs.
